# Protective Effects of Sacubitril/Valsartan on Cardiac Fibrosis and Function in Rats With Experimental Myocardial Infarction Involves Inhibition of Collagen Synthesis by Myocardial Fibroblasts Through Downregulating TGF-β1/Smads Pathway

**DOI:** 10.3389/fphar.2021.696472

**Published:** 2021-05-31

**Authors:** Meifang Wu, Yanguang Guo, Ying Wu, Kaizu Xu, Liming Lin

**Affiliations:** Department of Cardiology, Affiliated Hospital of Putian University, Southern Medical University, Putian, China

**Keywords:** sacubitril/valsartan, myocardial infarction, myocardial fibrosis, transforming growth factor-β1, myocardial fibroblasts

## Abstract

**Objectives:** To investigate the effect and mechanism of sacubitril/valsartan on myocardial fibrosis in rats following experimental myocardial infarction and in TGF-β1-treated myocardial fibroblasts.

**Methods:** Male Sprague-Dawley (SD) rats were subjected to coronary artery ligation to establish myocardial infarction and intragastrically fed vehicle, valsartan (Val, 32 mg/kg, once-daily) or sacubitril/valsartan (Sac/Val, 68 mg/kg, once-daily) for 4 weeks. In parallel, myocardial fibroblasts (MFs) isolated from neonatal SD rats were exposed to hypoxia and treated with TGF-β1 (5 ng/ml) plus vehicle, Val (10^7^–10^–5^ M) or Sac/Val (10^7^–10^5^ M). Rat cardiac function and fibrosis were measured by echocardiography and histological method, respectively. MFs viability and collagen synthesis were determined by cell counting kit-8 and enzyme-linked immunosorbent assay, respectively. Protein expressions of TGF-β1, Smad3, phosphorylated Smad3 (p-Smad3), and p-Smad3 subcellular localization were detected by immunoblotting and immunocytochemistry.

**Results:** Sac/Val significantly improved cardiac structure and function in rats after myocardial infarction, including decreased left ventricular end-diastolic diameter and interventricular septal thickness, increased ejection fraction, and reduced myocardial collagen volume fraction and type Ⅰ and type Ⅲ collagen levels, and this effect was superior to that of Val. Besides, Sac/Val inhibited myocardial TGF-β1 and p-Smad3 protein expression better than Val. Mechanically, Sac/Val significantly attenuated TGF-β1-induced proliferation and collagen synthesis of MFs, and inhibit Smad3 phosphorylation and nucleus translocation, and this effect outperformed Val. Overexpression and silencing of Smad3 enhanced and reversed the inhibitory effects of Sac/Val on TGF-β1-induced collagen synthesis by MFs, respectively.

**Conclusions:** Sacubitril/valsartan improves cardiac function and fibrosis in rats after experimental myocardial infarction, and this effect is related to the inhibition of collagen synthesis in myocardial fibroblasts by inhibiting the TGF/Smads signaling pathway.

## Introduction

Cardiovascular disease, especially ischemic heart disease, remains the leading cause of death around the world, accounting for 30% of all-cause deaths (Roth et al., 2017). Although early reperfusion therapy for acute myocardial infarction (MI) can effectively salvage ischemic myocardium, a considerable portion of cardiomyocytes may still occur irreversible necrosis and loss, followed by ventricular remodeling and cardiac insufficiency, which compromises the long-term survival of patients with myocardial infarction. Therefore, it is of particular importance to reverse ventricular remodeling post-MI to preserve cardiac function.

Sacubitril/valsartan, a first-in-class angiotensin receptor neprilysin inhibitor, is comprised of the angiotensin receptor blocker (ARB) valsartan and the neprilysin inhibitor pro-drug sacubitril in a 1:1 M ratio (Vardeny et al., 2013). Mechanistically, sacubitril/valsartan exerts its effects of diuresis, natriuresis, vasodilation, and neurohumoral regulation by simultaneous renin-angiotensin-aldosterone system blockade and natriuretic peptide system augmentation (Lin et al., 2016; Menendez 2016). In the landmark clinical trial of PARADIGM-HF, sacubitril/valsartan was demonstrated to outperform angiotensin-converting enzyme inhibitor (ACEI) in reducing heart failure hospitalization and cardiovascular death (McMurray et al., 2014). Accordingly, sacubitril/valsartan has been consistently recommended by national guidelines as a preferred treatment for heart failure with reduced ejection fraction to further reduce mortality and heart failure hospitalization when patients were still symptomatic after giving ACEI or ARB treatment (Ponikowski et al., 2016; Yancy et al., 2016; Maddox et al., 2021). However, there is scarce data regarding the effect and mechanism of sacubitril/valsartan in the setting of acute myocardial infarction (von Lueder et al., 2015; Ishii et al., 2017; Torrado et al., 2018).

Adverse ventricular remodeling is directly related to heart failure. The main causes of ventricular remodeling after myocardial infarction are compensatory myocardial hypertrophy in the noninfarcted area and myocardial fibrosis in the infarcted area (Sutton and Sharpe 2000). Current studies suggest that transforming growth factor-β1 (TGF-β1)/Smads signaling pathways play a pivotal role in the pathogenesis of post-infarction remodeling (Bujak and Frangogiannis 2007; Chen and Xie 2012; Khalil et al., 2017). Upon TGF-β receptor activation, Smad2 and Smad3 become phosphorylated and form heteromeric complexes with Smad4. These complexes translocate to the nucleus where they control gene expression that is involved in the fibrosis to amplify the fibrotic response (Petrov et al., 2002; Ma et al., 2018). Nevertheless, there is currently little literature on whether the effect of sacubitril/valsartan on myocardial fibrosis is related to its inhibition of the TGF-β1/Smads pathway.

Therefore, we aimed to investigate whether sacubitril/valsartan can improve cardiac remodeling and function in rats after myocardial infarction, and inhibit TGF-β1-induced myocardial fibroblast proliferation and collagen synthesis, and verify whether these effects are related to its inhibition of TGF-β1/Smads signaling pathway activation.

## Methods

### Establishment of Rat MI Model and Grouping

Male Sprague-Dawley (SD) rats (250 ± 20 g body weight) were used for experimental MI model establishment. MI was induced by ligation of the left anterior descending artery as previously described (Michael et al., 1995). Sham operation was performed by a similar manipulation without ligation. The mortality rate in 24 h was about 20%. Twenty-four surviving MI rats were randomly divided into three groups (*n* = 8 in each group): MI rats treated with vehicle (equal volume of normal saline, MI), MI rats treated with valsartan 32 mg/kg per day (MI-Val), and MI rats treated with sacubitril/valsartan 68 mg/kg per day (MI-Sac/Val). Eight sham-operated control rats (Sham) were also treated with vehicle. All treatment regimens were initiated 24 h following operation by gavage and continued for 4 weeks. All animals were housed under pathogen-free conditions, with free access to water and food and a 12 h light/12 h dark cycle. All animal protocols were reviewed and approved by the Animal Care and Use Committee of Affiliated Hospital of Putian University.

### Echocardiographic Measurements

Four weeks after treatment, transthoracic echocardiography was performed under anesthesia with pentobarbital sodium (40 mg/kg, i.p.) using a 10S (5.4–11.8 MHz) transducer (Vivid 7, GE Healthcare, Diegem, Belgium) by an echocardiography specialist. Two-dimensional M-mode measurements in short-axis view at the mid-papillary level include left ventricular end-diastolic dimension (LVEDd), left ventricular end-systolic dimension (LVEDs), interventricular septal wall thickness in diastole (IVSd), and left ventricular posterior wall thickness in diastole (LVPWd). The left ventricular ejection fraction (LVEF) and fractional shortening (FS) were automatically calculated by the echocardiographic system. All measurements were averaged over three consecutive cardiac cycles.

### Quantification of Myocardial Fibrosis

Picrosirius red staining was used for evaluating the fibrosis status of myocardial tissue. 4 μm paraffin sections from the mid-LV were stained with 0.1% Sirius red (Sigma Aldrich, St. Louis, MO, United States) in saturated picric acid (picrosirius red) for 1 h and mounted. Collagen volume fraction (CVF) of the peri-infarct zone was calculated as the ratio of stained area to that of the whole myocardial tissue using the computer-imaging software Image-Pro Plus 6.0.

### Cardiac Fibroblast Culture and Grouping

Myocardial fibroblasts (MFs) were isolated from neonatal SD rats (1–3 days old) as described previously (Chen et al., 2014). In brief, rats were killed, and the hearts were removed quickly under sterile conditions. Ventricular tissue was finely minced and placed in 0.25% trypsin for 15 min. Cell suspensions were plated in cell culture dishes for 90 min; most MFs adhered to the dishes. Nonadherent cells were removed. Cells were identified for positivity for vimentin. For induction of cellular hypoxia, the MFs were placed in a hypoxic chamber at 37°C with 94%N_2_, 5%CO_2_, and 1%O_2_ (Riches et al., 2009). MFs between passages 3–6 were used for experiment and grouped as follows: Control (MFs without any treatment), TGF-β1 (MFs treated with TGF-β1 5 ng/ml alone), Val (MFs treated with TGF-β1 5 ng/ml plus valsartan), and Sac/Val (MFs treated with TGF-β1 5 ng/ml plus sacubitril/valsartan). 50 ng/ml TGF-β1 stock solution was dissolved in DMEM and valsartan and sacubitril/valsartan stock solution was dissolved in DMEM with 0.1%DMSO.

### Cell Viability Assay

After each treatment, Cell Counting Kit-8 (Sigma) was used to measure the viability of MFs according to the standard protocol of manufacturer. The absorbance was determined at 450 nm using a spectrophotometer (Termo Fisher Scientifc, Waltham, MA, United States).

### Enzyme-Linked Immunosorbent Assay

Type Ⅰ and type Ⅲ collagen levels in infarcted myocardium and conditioned media of MFs were determined by enzyme-linked immunosorbent assay (ELISA) according to ELISA Kit protocols. For measurement of type Ⅰ and type Ⅲ collagen levels in supernatant of culture cells, MFs at passages 3–6 were seeded in 96-well plate (2,000 cells/well). Upon reaching 60–70% confluence, cells were replaced with 0.2%FBS-DMEM for 24 h, then pretreated with valsartan or sacubitril/valsartan (10^7^–10^5^ M) for 45 min, followed by incubation with TGF-β1 (5 ng/ml) for 48 h. The cell supernatant was collected, and the type Ⅰ and type Ⅲ collagen contents were calculated.

### Cellular Fractionation and Immunoblotting

The NE-PER™ Nuclear and Cytoplasmic Extraction Reagents kit (ThermoFisher, United States) was utilized to extract nuclear and cytosolic protein fractions (Cao et al., 2004). GAPDH and TFIIA-α were used to control equal loading of cytoplasmic and nuclear fractions, respectively. For immunoblotting, the corresponding proteins were electrophoresed on 10% SDS-PAGE and transferred onto nitrocellulose membranes. The membranes were blocked with 5% dry milk in PBS-Tween buffer (0.1% Tween 20; pH7.5) for 60 min and incubated with primary antibodies to TGF-β1 (Santa Cruz, CA, United States), Smad3, and p-Smad3 (Ser423/425) (Cell Signaling Technology, Beverly, MA, United States), respectively, for 3 h at room temperature. After five washes with TBST and two washes with TBS, the membrane was incubated for 1 h at room temperature with horseradish peroxidase-conjugated secondary antibody (Abcam). Following another two washes with TBST, labeled proteins were visualized using ECL (Invitrogen, Carlsbad, CA) on high-performance chemiluminescence film. The intensity of the bands was quantified by densitometry with image analysis software. Smad3 nucleus translocation was determined as the percentage of protein content in the nucleus *versus* cytoplasm.

### Immunocytochemical Staining

Subcellular localization of p-Smad3 was detected by immunocytochemical staining. After treatments, cells were stained with 1:100 p-Smad3 polyclonal antibody (Cell Signaling Technology, Beverly, MA, United States) using the SABC immunocytochemical method according to the manufacturer’s instructions (Huang et al., 2007).

### Plasmid-DNA Transient Transfection

Cells in exponential growth phase were plated in 6-well plates at a density of 1 × 10^5^ cell/well. When 60–70% confluence was reached, transient transfection was performed. 0.8 µg plasmid and 2 µl liposome were added into 500 µl OPTI-MEM media, respectively, and gently mixed. After incubated at room temperature for 5 min, OPTI-MEM media containing Smad3 DNA plasmid and liposome was mixed and incubated for another 30 min. For transfection, 1 ml liposome-Smad3-plasmid -DNA-OPTI-MEM media complex was added into each well. For the blank control group, another 1 ml OPTI-MEM media was added; for the liposome control group, 1 ml liposome-OPTI-MEM media complex was added. After 24 h, the transfection medium was replaced by a serum-free M199 medium for subsequent experiments.

### SiRNA Transient Transfection

Smad3 small interfering RNA (siRNA) and control siRNA were purchased from Sigma Biotechnology (Sigma, United States). The siRNAs were transfected into MFs according to the manufacturer’s instructions using the Lipofectamine®RNAiMAX transfection reagent (Invitrogen, United States). MFs between passages 3–6 were isolated with 0.25% Tyrisin and seeded in 6-well plates at a density of 1 × 10^5^cells/well containing M199 with 10%FBS. When cells grew to 60–70% confluence, the medium was replaced by serum-free M199 for 24 h and incubated with 50 nM control or Smad3 siRNA for 6 h in serum-free OPTI-MEM media (Invitrogen, United States). After the incubation, the transfected media was replaced by a serum-free M199 medium for subsequent experiments.

### Statistical Analysis

Data are presented as mean ± SD. One-way ANOVA and multiple comparisons were performed using GraphPad Prism 6.0 (GraphPad Software, San Diego, CA). Tukey’s post-test was used to determine differences between the groups. *P* values less than 0.05 were considered significant.

## Results

### Effects of Sacubitril/Valsartan on Cardiac Structure and Function in Rats After Myocardial Infarction

**FIGURE 1:**
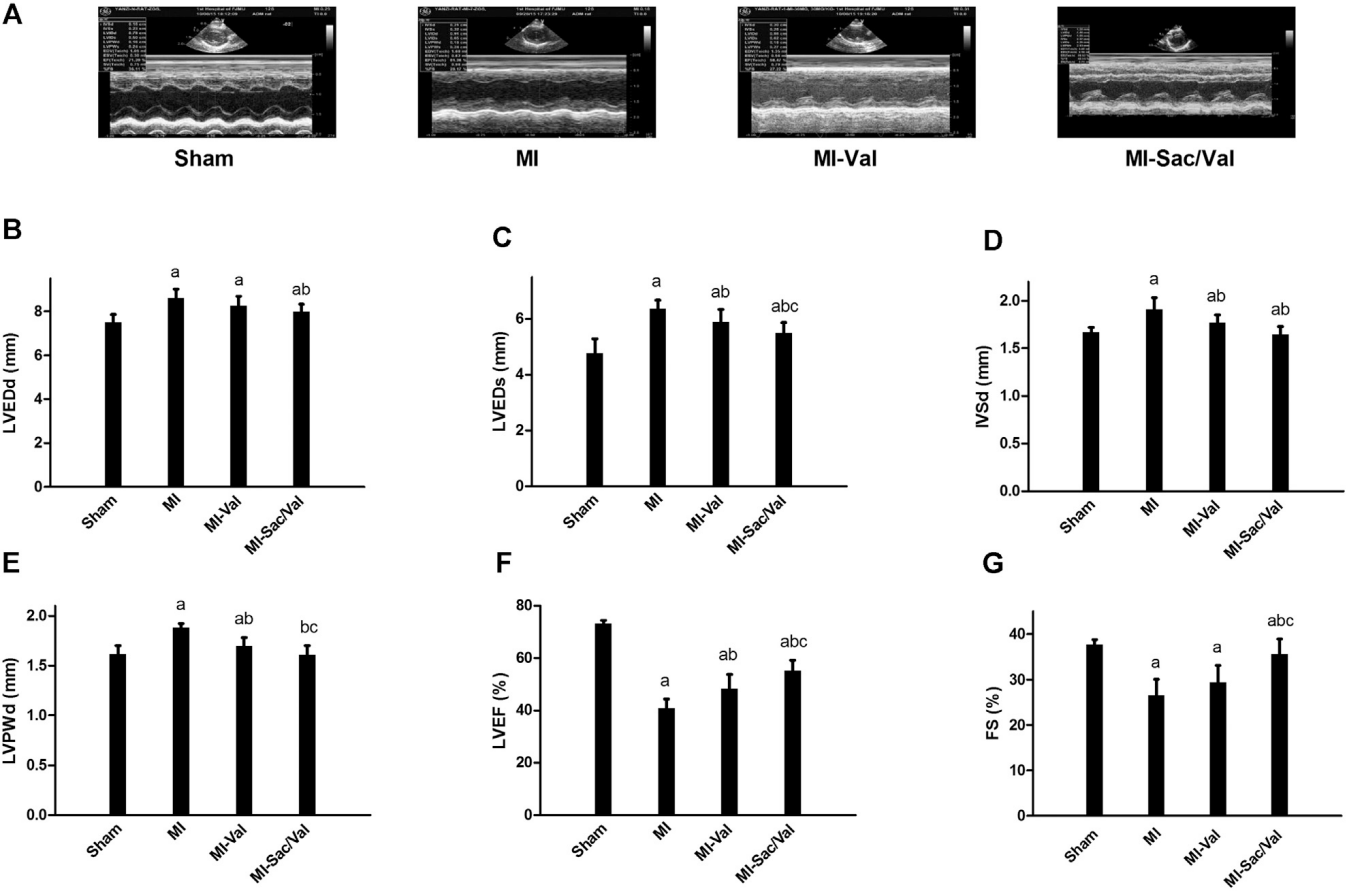
Effects of sacubitril/valsartan on cardiac structure and function determined by echocardiogary in rats with experimental MI. **(A)** Representative images of M-mode echocardiography. **(B–G)** Quantitative measurement of echocardiographic parameters. LVEDd, left ventricular end-diastolic dimension; LVEDs, left ventricular end-systolic dimension; IVSd, interventricular septal thickness in diastole; LVPWd, left ventricular posterior wall thickness in diastole; LVEF, left ventricular ejection fraction; FS, fractional shortening; MI, myocardial infaction; Val, valsartan; Sac/Val, sacubitril/valsartan. Results are mean ± SD (*n* = 8); a*P* < 0.05 *vs.* Sham; b*P* < 0.05 *vs.* MI; c*P* < 0.05 *vs.* MI-Val.

Echocardiography showed that rats following myocardial infarction establishment exhibited significantly increased LVEDd, LVEDs, IVSd, and LVPWd (*p* < 0.05), but significantly decreased LVEF and FS (*p* < 0.05), compared with sham rats. Sacubitril/valsartan treatment reversed the above adverse change of cardiac structure and function induced by myocardial infarction, and this effect was superior to the traditional heart failure drugs valsartan, which was characterized by a further significant improvement in LVEDs, LVPWd, LVEF, and FS (*p* < 0.05), and a non-significant decrease trend in LVEDd and IVSd (*p* > 0.05) (Figure 1).

### Effects of Sacubitril/Valsartan on Ventricular Remodeling in Rats After Myocardial Infarction

**FIGURE 2:**
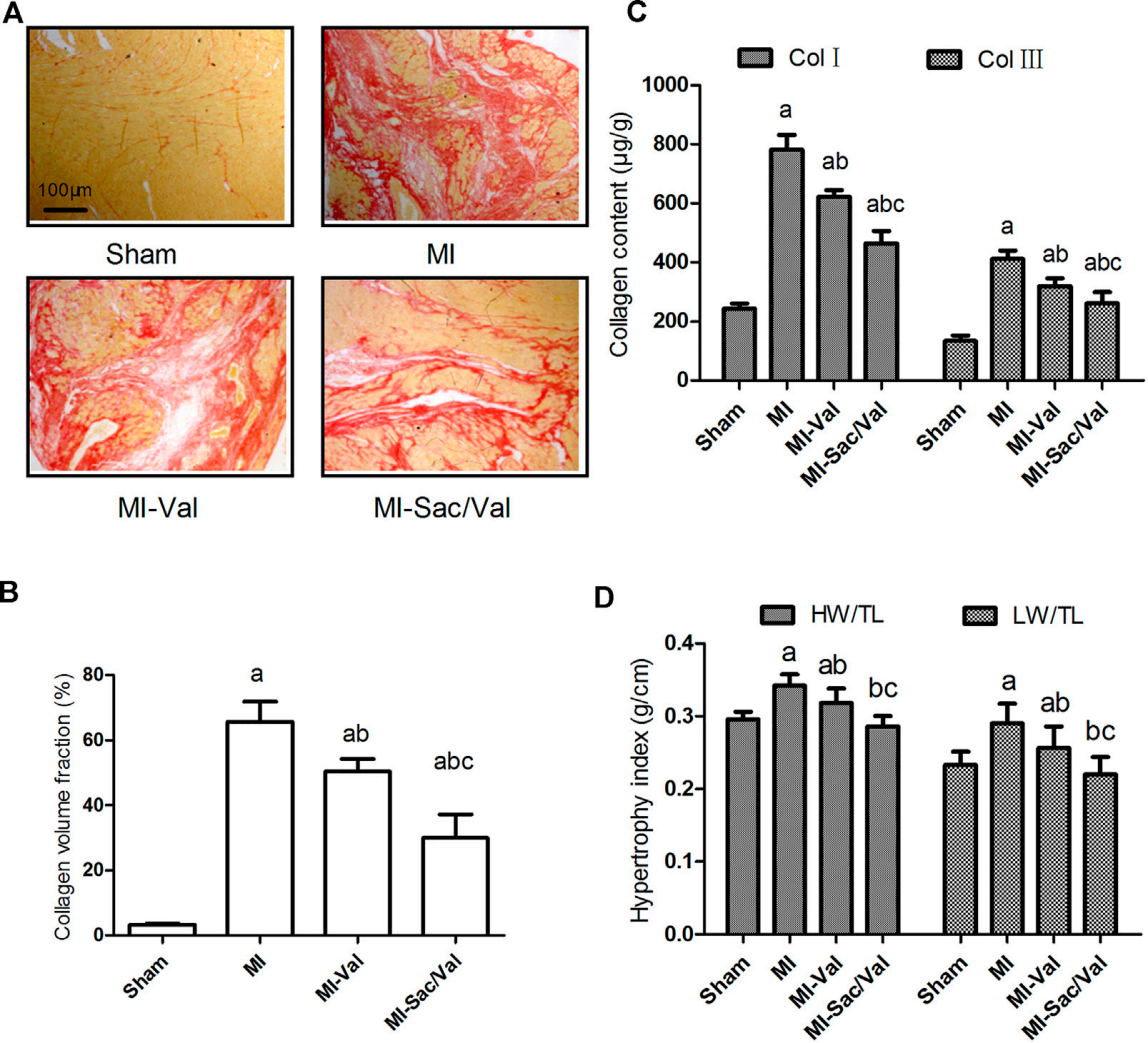
Effects of sacubitril/valsartan on ventricular remodeling in rats after myocardial infarction. **(A)** Representative photomicrographs of collagen volume in the infarcted myocardium (Picrosirius red staining, scale bars = 100 μm). **(B)** Myocardial collagen volume fraction quantified by digital planimetry. **(C)** Myocardial type Ⅰ and type Ⅲ collagen content detemined by ELISA. **(D)** Myocardial hypertrophy expressed as heart weight to tibia length ratio (HW/TL) and left ventricular weight to tibia length ratio (LW/TL). Results are mean ± SD (*n* = 8); Col Ⅰ, type Ⅰ collagen; Col Ⅲ, type Ⅲ collagen; HW/TL, heart weight to tibia length ratio; LW/TL, left ventricular weight to tibia length ratio; MI, myocardial infaction; Val, valsartan; Sac/Val, sacubitril/valsartan. Results are mean ± SD (*n* = 8); a*P* < 0.05 *vs.* Sham;b *P* < 0.05 *vs.* MI;c*P* < 0.05 *vs.* MI-Val.

Picrosirius red staining showed that only a small amount of filamentous red staining was found in the myocardium of sham rats, while diffuse patchy red staining was found in the infarcted myocardium of MI rats. Small bundles of diffuse red-stained fibers were observed in the infarcted myocardium of rats receiving valsartan treatment, while only fine bundles of red-stained fibers were observed in the infarcted myocardium of rats receiving sacubitril/valsartan treatment (Figure 2A). The collagen content of rats in each group was then compared semi-quantitatively by calculating CVF. The results showed that the CVF of MI rats was dramatically increased compared with that of sham rats (*p* < 0.05). Both sacubitril/valsartan and valsartan treatment remarkably blunted the increased CVF observed in MI rats, and sacubitril/valsartan had a significantly better effect than valsartan, as shown in Figure 2B.

Consistent with the difference of myocardial CVF between groups, the type Ⅰ and type Ⅲ collagen content in infarcted myocardium of MI rats were significantly higher than those of sham rats (*p* < 0.05). Both sacubitril/valsartan and valsartan treatment effectively reduced the content of type Ⅰ and type Ⅲ collagen in infarcted myocardium (*p* < 0.05), and sacubitril/valsartan had a better effect than valsartan (*p* < 0.05) (Figure 2C).

Compensatory cardiac hypertrophy in non-infarct areas is also an important feature of ventricular remodeling after MI, and cardiac hypertrophy is reflected by calculating the ratio of the whole heart or left ventricular weight to tibial length. The results showed that the myocardial hypertrophy index of MI rats was significantly higher than that of sham rats (*p* < 0.05). Both sacubitril/valsartan and valsartan effectively inhibited the myocardial hypertrophy of MI rats (*p* < 0.05), and the effect of sacubitril/valsartan was superior to that of valsartan (*p* < 0.05), with the myocardial hypertrophy index of sacubitril/valsartan-treated rats similar to that of sham rats (*p* > 0.05) (Figure 2D).

### Effects of Sacubitril/Valsartan on Myocardial TGF-β1, Smad3 and p-Smad3 Protein Expression in Rats After Myocardial Infarction

**FIGURE 3:**
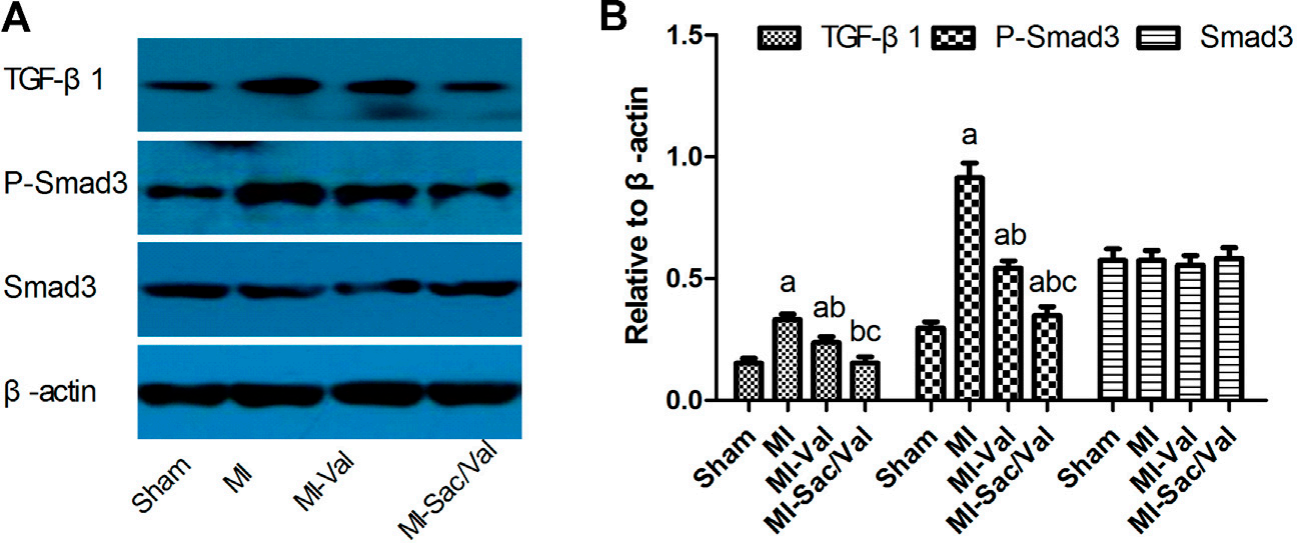
Effects of sacubitril/valsartan on myocardial TGF-β1, Smad3 and p-Smad3 protein expression in rats after myocardial infarction. **(A)** Representive immunoblots of protein TGF-β1, p-Smad3 and Smad3 in infarcted myocardium. **(B)** Bars represent protein quantification of TGF-β1, p-Smad3 and Smad3 relative to β-actin. TGF-β1, transforming growth factor-β1; MI, myocardial infaction; Val, valsartan; Sac/Val, sacubitril/valsartan. Results are mean ± SD (*n* = 8); a*P* < 0.05 *vs.* Sham; b*P* < 0.05 *vs.* MI; c*P* < 0.05 *vs.* MI-Val.

Immunoblotting analysis showed that the protein expression levels of TGF-β1 and p-Smad3 in the infarcted myocardium of MI rats were significantly higher than those of sham rats (*p* < 0.05). Both sacubitril/valsartan and valsartan treatment remarkably abrogated the upregulated protein expression of TGF-β1 and p-Smad3 observed in infarcted myocardium of MI rats, and the inhibitory effect of sacubitril/valsartan was significantly better than that of valsartan (*p* < 0.05). There was no significant difference in the expression of Smad3 protein in the myocardium among all groups (*p* > 0.05) (Figure 3).

### Effects of Sacubitril/Valsartan on TGF-β1 -Induced Collagen Synthesis in Myocardial Fibroblasts Under Hypoxia Conditions

**FIGURE 4:**
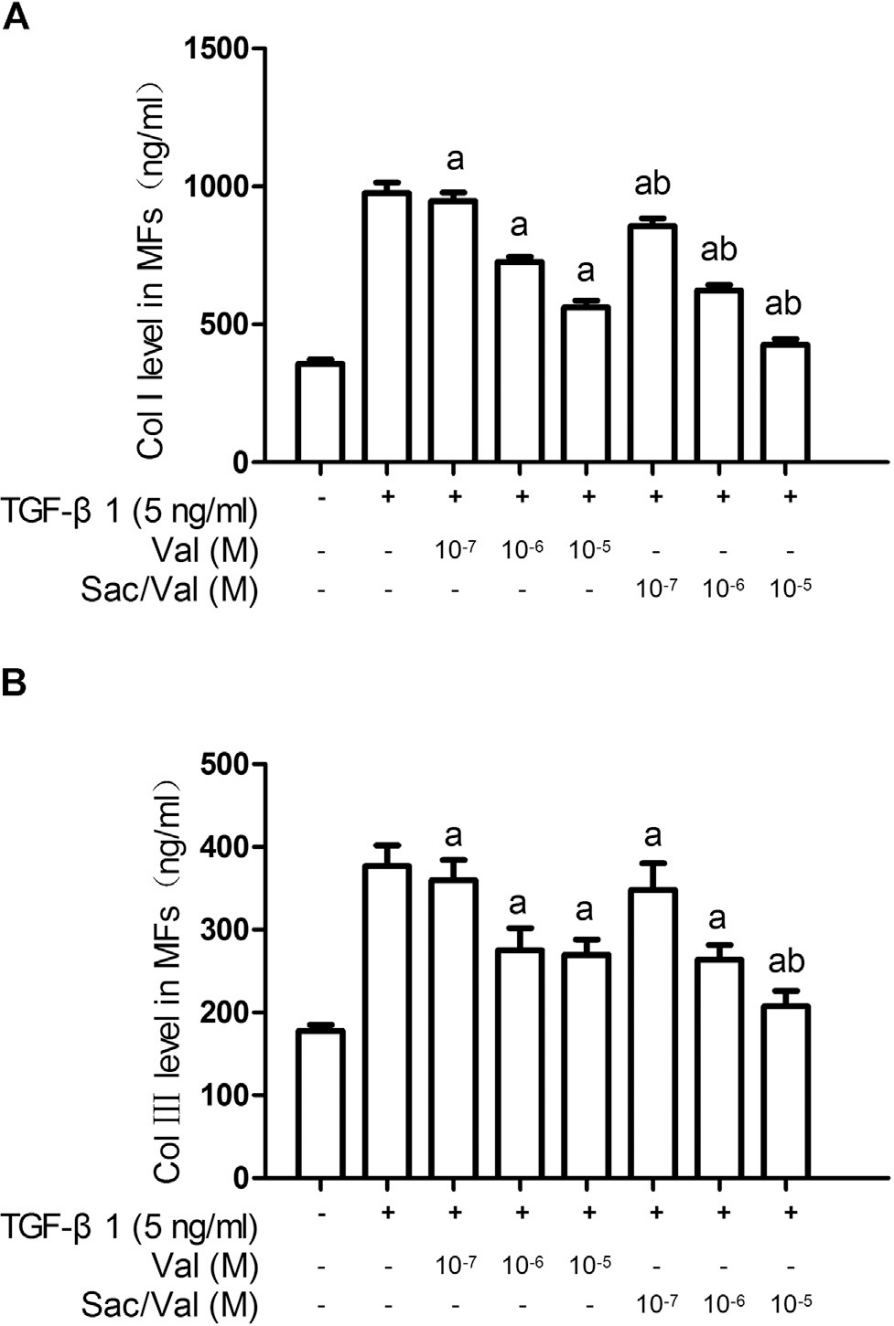
Effects of sacubitril/valsartan on TGF-β1-induced collagen synthesis in myocardial fibroblasts under hypoxia conditions. MFs were inoculated in 96-well plates and replaced with 0.2%FBS when the cells grew to 60–70% confluence for 24 h. After sacubitril/valsartan (10^7^–10^5^ M) or valsarten (10^7^–10^5^ M) was added for 45 min, TGF-β1 (5 ng/ml) was added for co-incubation in hypoxia environment for 48 h. The supernatant was collected and collagen levels were measured by ELISA. **(A)** and **(B)** represent type Ⅰ and Ⅲ collagen levels in myocardial fibroblasts, respectively. Col Ⅰ, type Ⅰ collagen; Col Ⅲ, type Ⅲ collagen; MFs, myocardial fibroblasts; TGF-β1, transforming growth factor-β1; Val, valsartan; Sac/Val, sacubitril/valsartan. Results are mean ± SD (*n* = 8); a*P* < 0.05 *vs.* TGF-β1 (5 ng/ml); b*P* < 0.05 *vs.* equivalent moles of Val.

ELISA results showed that TGF-β1 (0.1–10 ng/ml) promoted collagen synthesis in MFs under hypoxia conditions in a concentration-dependent manner, and this effect reached its peak at 5 ng/ml and 10 ng/ml of TGF-β1 (*p* < 0.05) (Supplementary Figure S1). Both sacubitril/valsartan (10^7^–10^5^ M) and valsartan (10^7^–10^5^ M) dose-dependently inhibited type Ⅰ and type Ⅲ collagen synthesis in MFs induced by TGF-β1 (5 ng/ml) under hypoxia condition, that is, the collagen synthesis by MFs was most significantly inhibited when sacubitril/valsartan and valsartan were at a concentration of 10^5^ M (*p* < 0.05), and this concentration was therefore used as the working concentration of subsequent cell experiments. Compared with the equivalent moles of valsartan, the type Ⅰ collagen level in MFs treated with sacubitril/valsartan (10^7^–10^5^ M) was further significantly decreased. Similarly, the type Ⅲ collagen level in MFs treated with sacubitril/valsartan 10^7^ and 10^6^ M showed a non-significant decrease trend, while sacubitril/valsartan 10^5^ M showed a further significant decrease compared with the equivalent moles of valsartan (*p* < 0.05) (Figure 4).

### Effects of Sacubitril/Valsartan on TGF-β1 -Induced Increased Cell Viability of Myocardial Fibroblasts Under Hypoxia Conditions

**FIGURE 5:**
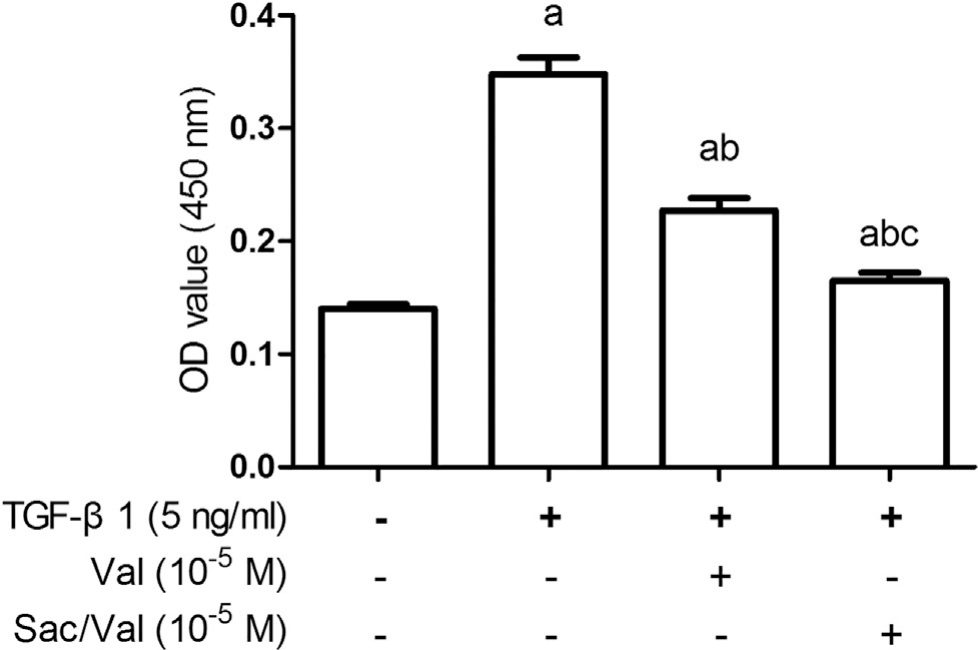
Effects of sacubitril/valsartan on TGF-β1-induced increased viability of myocardial fibroblasts under hypoxia conditions. MFs were inoculated in 96-well plates and replaced with 0.2%FBS when the cells grew to 60–70% confluence for 24 h. After sacubitril/valsartan (10^5^ M) or valsarten (10^5^ M) was added for 45 min, TGF-β1 (5 ng/ml) was added for co-incubation in hypoxia environment for 48 h. Cell viability of MFs was detected by CCK-8 assay. OD, optical density; TGF-β1, transforming growth factor-β1; Val, valsartan; Sac/Val, sacubitril/valsartan. Results are mean ± SD (*n* = 8); a*P* < 0.05 *vs.* TGF-β1 (0 ng/ml); b*P* < 0.05 *vs.* TGF-β1 (5 ng/ml); c*P* < 0.05 *vs.* Val.

The viability of MFs was detected by CCK-8 method, and the results showed that the cell viability of MFs in the TGF-β1 (5 ng/ml) group was significantly increased compared with control MFs (TGF-β1 0 ng/ml) (*p* < 0.05). Both sacubitril/valsartan (10^5^ M) and valsartan (10^5^ M) pretreatment markedly mitigated the increased cell viability of MFs induced by TGF-β1 (5 ng/ml) (*p* < 0.05). Similarly, the inhibitory effect of sacubitril/valsartan(10^5^ M) on MFs viability significantly outperformed valsartan (10^5^ M) (Figure 5).

### Effects of Sacubitril/Valsartan on TGF-β1-Induced Smad3 Phosphorylation and Nucleus Translocatoin in Myocardial Fibroblasts Under Hypoxia Conditions

**FIGURE 6:**
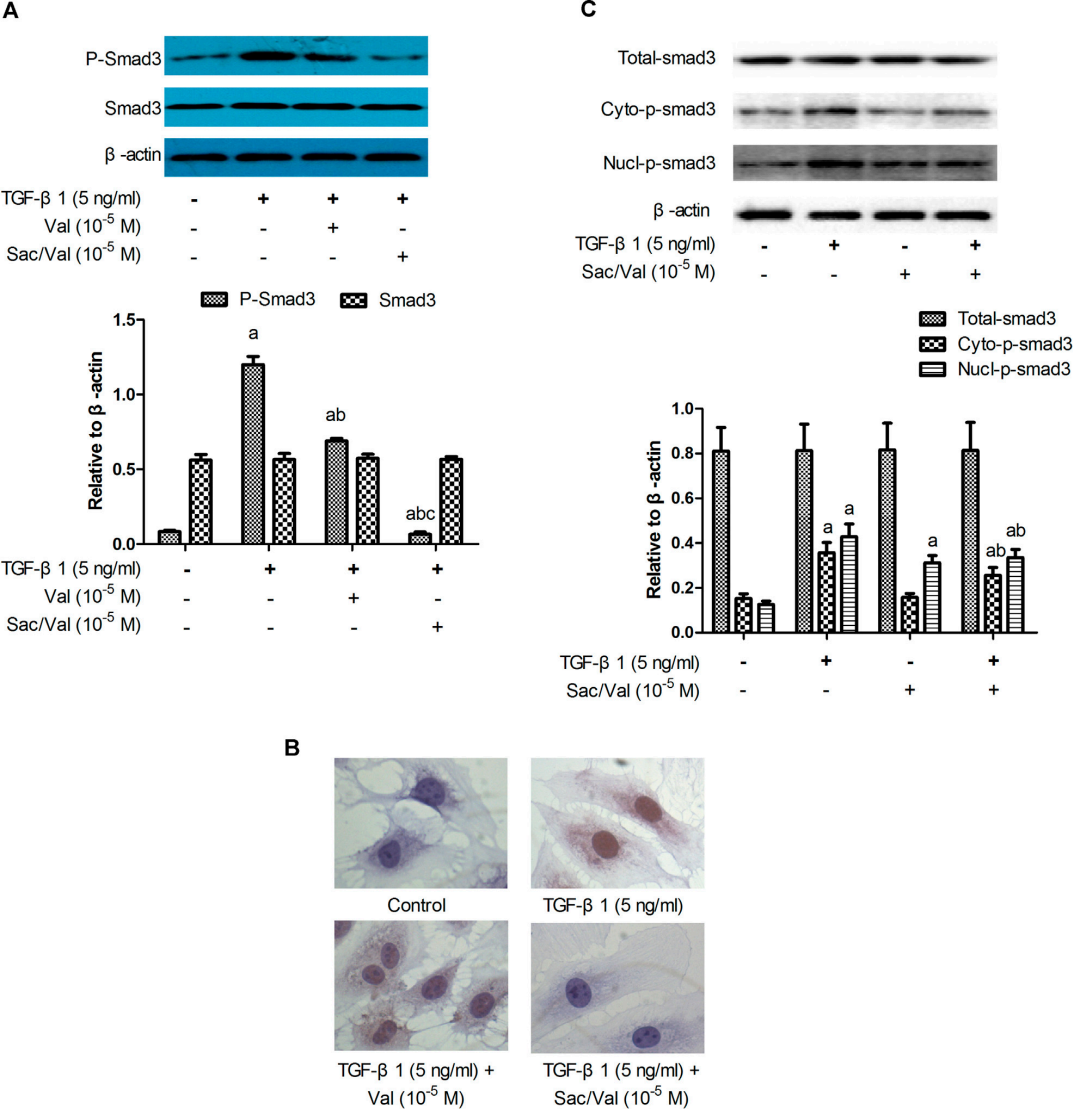
Effects of sacubitril/valsartan on TGF-β1 -induced Smad3 phosphorlation and nucleus translocatoin in myocardial fibroblasts. MFs were pretreated with sacubitril/valsartan (10^5^ M) or valsartan (10^5^ M) for 45  min before adding TGF-β1 (5 ng/ml) for 60 min **(A)** Smad3 phosphorlation determined by immunoblotting. **(B)** Smad3 subcellular localization detected by immunocytochemistry (objective 40×). **(C)** Smad3 subcellular localization demonstrated by immunoblotting. TGF-β1, transforming growth factor-β1; Val, valsartan; Sac/Val, sacubitril/valsartan; Cyto-p-Smad3, phosphorylated Smad3 in the cytoplasm; Nucl-p-Smad3, phosphorylated Smad3 in the nucleus; Results are mean ± SD (n = 3); a*P* < 0.05 *vs.* TGF-β1 (0 ng/ml); b*P* < 0.05 *vs.* TGF-β1 (5 ng/ml); c*P* < 0.05 *vs.* Val.

The results of immunoblotting showed that TGF-β1 (5 ng/ml) treatment upregulated the p-Smad3 protein expression in MFs under hypoxia conditions in a time-dependent manner (Supplementary Figure S2). The p-Smad3 protein level was dramatically increased after 45 min of TGF-β1 treatment (*p* < 0.05) and reached the peak at 60 min. Both sacubitril/valsartan (10^5^ M) and valsartan (10^5^ M) significantly reduced the TGF-β1-induced upregulation of p-Smad3 protein expression (*p* < 0.05), and consistently sacubitril/valsartan (10^5^ M) showed a better inhibitory effect compared with valsartan (10^5^ M) (*p* < 0.05). There was no significant change in Smad3 protein levels among all groups (*p* > 0.05) (Figure 6A).

Immunocytochemical staining was performed to evaluate the p-Smad3 subcellular localization. In control MFs, pale brown granules indicating p-Smad3 were not observed in the cytoplasm and nucleus; In MFs treated with TGF-β1, massive pale brown granules were observed in the cytoplasm and nucleus. In MFs treated with TGF-β1 plus valsartan, less number of pale brown granules were observed. In MFs treated with TGF-β1 plus sacubitril/valsartan, few pale brown granules were observed in the cytoplasm and nucleus (objective 40×) (Figure 6B). The protein expression levels of cytoplasmic and nuclear Smad3 were detected by immunoblotting to further address the effect of sacubitril/valsartan on p-Smad3 subcellular localization. The results showed that TGF-β1 slightly and nonsignificantly increased the cytoplasmic p-Smad3 level of MFs (*p* > 0.05), but markedly increased the nuclear Smad3 expression compared with the control MFs (*p* < 0.05). Sacubitril/valsartan (10^5^ M) pretreatment significantly reduced the cytoplasmic and nuclear p-Smad3 protein expression compared with TGF-β1 treated MFs (*p* < 0.05). There was no significant difference in Smad3 levels among all groups (*p* > 0.05) (Figure 6C).

### Smad3 Mediated the Inhibitory Effects of Sacubitril/Valsartan on TGF-β1 -Induced Collagen Synthesis in Myocardial Fibroblasts Under Hypoxia Conditions

**FIGURE 7:**
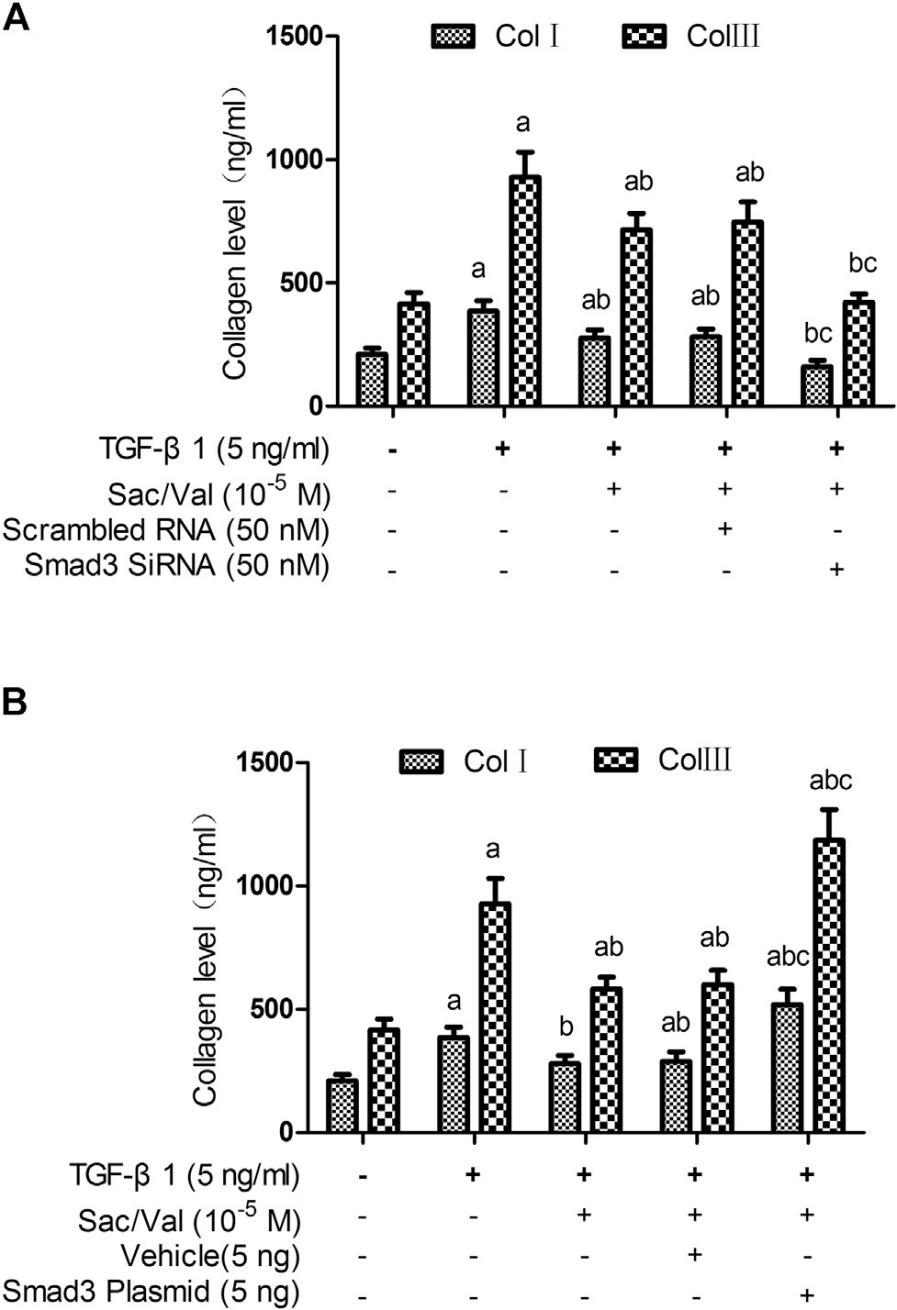
Effect of Smad3 silencing and overexpression on the inhibitory effect of sacubitril/valsartan on TGF-β1 -induced collagen synthesis by myocardial fibroblasts under hypoxia conditions. MFs were transfected with Smad3-specific siRNA or a plasmid encoding for Smad3 to induce Smad3 silencing **(A)** and overexpression **(B)**, respectively, then the transfected cells were treated with sacubitril/valsartan (10^5^ M) for 45 min, followed with TGF-β1 (5 ng/ml) co-incubation in hypoxia environment for 48 h. The supernatant was collected and collagen levels were measured by ELISA. Col Ⅰ, type Ⅰ collagen; Col Ⅲ, type Ⅲ collagen; TGF-β1, transforming growth factor-β1; Sac/Val, sacubitril/valsartan. Results are mean ± SD (*n* = 6); a*P* < 0.05 *vs.* TGF-β1 (0 ng/ml); b*P* < 0.05 *vs.* TGF-β1 (5 ng/ml); c*P* < 0.05 *vs.* TGF-β1 (5 ng/ml) + sacubitril/valsartan (10^5^ M).

To evaluate the effect of Smad3 silencing on the inhibitory effect of sacubitril/valsartan on TGF-β1-induced collagen synthesis by MFs under hypoxia conditions, we transfected the MFs with Smad3-specific siRNA. Immunoblotting analysis showed that the expression of Smad3 protein was significantly decreased after MFs transfected with Smad3-specific siRNA (25 and 50 nM) compared with the scramble group (*p* < 0.05) and was dose-dependent (25 *vs.* 50 nM, *p* < 0.05) (Supplementary Figure S3). When MFs were transfected with specific Smad3 siRNA (50 nM), the TGF-β1-induced p-Smad3 nuclear translocation was also significantly compromised (*p* < 0.05) (Supplementary Figure S4). Most importantly, Smad3 silencing enhanced the inhibitory effect of sacubitril/valsartan on TGF-β1-induced collagen synthesis by MFs under hypoxia conditions (*p* < 0.05) (Figure 7A).

To evaluate the effect of Smad3 overexpression on the inhibitory effect of sacubitril/valsartan on TGF-β1-induced collagen synthesis by MFs under hypoxia conditions, we transfected the MFs with Smad3 plasmid. Immunoblotting analysis showed that the expression of Smad3 protein was significantly increased after transfection of Smad3 plasmid into MFs (*p* < 0.05) (Supplementary Figure S5). When MFs were transfected with Smad3 plasmid, the TGF-β1-induced p-Smad3 nuclear translocation was also significantly increased (*p* < 0.05) (Supplementary Figure S6). Most importantly, Smad3 overexpression attenuated the inhibitory effect of sacubitril/valsartan on TGF-β1-induced collagen synthesis by MFs under hypoxia conditions (*p* < 0.05) (Figure 7B).

## Discussion

Based on the results of the PARADIGM-HF trial, sacubitril/valsartan is currently recommended by national guidelines as the preferred drug for the treatment of patients with heart failure with reduced ejection fraction compared to traditional heart failure drugs ACEI or ARB. However, it remains unclear whether it also has an advantage over ACEI or ARB in patients with acute myocardial infarction and the underlying mechanism. This study demonstrated *in vivo* that, sacubitril/valsartan outperformed valsartan in reducing collagen deposition in the infarcted region and compensatory hypertrophy in the noninfarcted region, and eventually further improved cardiac structure and function in rats following myocardial infarction compared to valsartan. Mechanistically, we further revealed *in vitro* that the inhibitory effects of sacubitril/valsartan on myocardial fibroblast proliferation and collagen synthesis through downregulation of TGF-β1/Smads signaling may be casually related to its better cardioprotective effects.Ventricular remodeling post-MI is known to be associated with the progression of heart failure and poor long-term prognosis. This pathological process mainly includes compensatory hypertrophy of myocardial cells in the noninfarcted area and reactive myocardial fibrosis in the infarcted area due to increased synthesis of collagen. In this study, we showed that sacubitril/valsartan treatment further reduced LVEDd and IVST and increased LVEF and FS in rats following myocardial infarction compared with valsartan. Histopathology revealed that sacubitril/valsartan significantly inhibited compensatory hypertrophy of the noninfarcted area (manifested as lower HMI and LVMI) and reduced collagen deposition in the peri-infarct area (manifested as lower CVF and type Ⅰ and type Ⅲ collagen level). These results were consistent with other studies investigating sacubitril/valsartan’s effects on post-MI fibrosis. Torrado et al. proved that sacubitril/valsartan was superior to valsartan in reducing left ventricular scar size and LVEF in rabbit model of myocardial infarction (Torrado et al., 2018). Von Lueder et al. found that sacubitril/valsartan was superior to ARB alone in reversing ventricular remodeling (including myocardial hypertrophy and fibrosis) in experimental myocardial infarction rats (von Lueder et al., 2015). Kompa, A. R, et al. reported that sacubitril/valsartan versus ACEI perindopril reduced myocardial tissue inhibitors of metalloproteinase-2 gene expression with a trend to lowering type Ⅰ collagen, although reductions in cardiac structural remodeling were similar (Kompa et al., 2018). However, these studies did not delve into the cellular and molecular mechanism of sacubitril/valsartan’s anti-remodeling effects.

Basic studies have found that overactivation of TGF-β1/Smads signaling pathway plays an important role in inducing and exacerbating the pathological process of myocardial fibrosis after myocardial infarction (Bujak and Frangogiannis 2007; Khalil et al., 2017; Walton et al., 2017). Specifically, Upon binding with TGF-β1 receptor on the surface of myocardial fibroblasts, TGF-β1 stimulates phosphorylation of downstream Smads protein (mainly Smad2/3) and translocation into the nucleus, induces myocardial fibroblasts proliferation, phenotypic transformation, and collagen synthesis, and ultimately promotes extracellular matrix formation and myocardial fibrosis (Petrov et al., 2002; Shen et al., 2011; Ma et al., 2018). However, to date, there are few reports on whether sacubitril/valsartan’s anti-myocardial fibrosis is related to its inhibition of the TGF-β1/Smads pathway. Our *in vivo* experiment showed that both sacubitril/valsartan and valsartan could effectively inhibit the expression of TGF-β1 and p-Smads in the infarcted area. Our results have been further confirmed by a recent study by Zhang W et al., who found in rats with heart failure with preserved ejection fraction induced by high-salt diet that, sacubitril/valsartan effectively alleviated the symptoms probably by inhibiting fibrosis *via* the TGF-β1/Smad3 signaling pathway (Zhang et al., 2020).

More important, our *in vivo* experiment results suggest a better inhibitory effect of sacubitril/valsartan than valsartan in inhibiting TGF-β1/Smads pathway activation. Compared with vehicle treatment, sacubitril/valsartan and valsartan reduced TGF-β1 protein expression by 71.7 and 42.3%, respectively, and reduced p-Smad3 protein expression by 61.9 and 40.6%, respectively, while imposed no significant effect on Smad3 protein expression. This results were further confirmed in our *in vitro* experiments in myocardial fibroblast under hypoxia conditions, which demonstrated that sacubitril/valsartan was superior to valsartan in inhibiting the viability and collagen synthesis of myocardial fibroblasts induced by TGF-β1, and in reducing Smad3 phosphorylation and nucleus translocalization. Overexpression and silencing of Smad3 reversed and enhanced the inhibitory effect of sacubitril/valsartan on collagen synthesis induced by TGF-β1 in myocardial fibroblasts, respectively. The better inhibition of the TGF-β1/Smad3 pathway by sacubitril/valsartan compared with valsartan was considered to be related to the neprilysin inhibition by sacubitril and increased natriuretic peptide levels. Li et al. has proven for the first time that atrial natriuretic peptide disrupts TGF-β1-induced phosphorylation and nuclear translocation of pSmad3 and downstream events, including myofibroblast transformation, proliferation, and expression of extracellular matrix molecules in myocardial fibroblasts, by increasing levels of cyclic guanosine monophosphate and phosphokinase G (PKG) (Li et al., 2008). We demonstrated both *in vivo* and *in vitro* that sacubitril/valsartan can inhibit myocardial fibrosis by antagonizing the TGF-β1/Smad pathway. However, how sacubitril/valsartan directly acts on myocardial fibroblasts in the *in vitro* experiment in the absence of natriuretic peptide, whether it is achieved by increasing intracellular PKG level of myocardial fibroblasts as Ryan M Burke, et al. suggested in a mouse model of left ventricle pressure overload (Burke et al., 2019), and whether elevated PKG also affected the TGF-β1/non-Smad-dependent pathways remains to be determined.

Also of note is that, sacubitril/valsartan was observed to inhibit myocardial fibrosis more significantly than its effects on myocardial TGF-β1 and p-Smads protein expression, which suggests that other mechanisms besides inhibition of the TGF-β1/Smads signaling pathway are involved in the cardioprotective effects of sacubitril/valsartan after myocardial infarction. In recent years, studies have found that sacubitril/valsartan can improve ventricular remodeling after myocardial infarction through other mechanisms, such as inhibiting the release of pro-inflammatory cytokines and the activity of matrix metalloproteinase-9, preventing antioxidant enzyme against degradation, and downregulating exosomal miR-181a (Ishii et al., 2017; Imran et al., 2019; Vaskova et al., 2020). More mechanistic studies are warranted to elucidate the cardioprotective effects of sacubitril/valsartan.

There exists several limitations in our study. First, the echocardiographic parameters of each group of rats at baseline were not measured and matched, especially the ejection fraction at baseline, which restricted the specific promotion of the experimental conclusions; Second, we did not detect plasma N-terminal pro-B-type natriuretic peptide level in rats, thus affecting comprehensive assessment of the cardioprotective effects of sacubitril/valsartan; Third, we only demonstrated that sacubitril/valsartan can alleviate TGF-β1-induced myocardial fibrosis through Smad-dependent pathway, but did not discuss the Smad-independent pathway, which needs to be further explored in future studies.

In conclusion, our study found that sacubitril/valsartan can better inhibit ventricular remodeling after myocardial infarction than valsartan and improve cardiac function, and this effect is related to inhibition of collagen synthesis by myocardial fibroblasts through downregulating TGF-β1/Smads pathway. However, it remains to be determined the superiority of sacubitril/valsartan in reducing heart failure events after MI in the forthcoming PARADISE-MI trial (Lefer and Sharp 2018).

## Data Availability

The original contributions presented in the study are included in the article/Supplementary Material, further inquiries can be directed to the corresponding authors.
