# Supplementary material for: Protective Effects of Sacubitril/Valsartan on Cardiac Fibrosis and Function in Rats With Experimental Myocardial Infarction Involves Inhibition of Collagen Synthesis by Myocardial Fibroblasts Through Downregulating TGF-β1/Smads Pathway
Source: Front Pharmacol. 2021 May 31;12:696472. doi: 10.3389/fphar.2021.696472 (PMC8201773; doi:10.3389/fphar.2021.696472)
Supplement: Supplementary file 1 [file DataSheet1.doc]

Supplementary figure 1 Dose-effect relationship of TGF-β1 on collagen synthesis in myocardial fibroblasts under hypoxia conditions. MFs were inoculated in 96-well plates and replaced with 0.2%FBS when the cells grew to 60%-70% confluence for 24 h. TGF-β1 (0.1-10 ng/ml) was added for co-incubation for 48 h. The supernatant was collected and type Ⅰ and type Ⅲ collagen levels were measured by ELISA. Col Ⅰ, type Ⅰ collagen; Col Ⅲ, type Ⅲ collagen; TGF-β1, transforming growth factor-β1. Results are mean ± SD; a*P* < 0.05 *vs.* TGF-β1 (0.1 ng/ml); b*P* < 0.05 *vs.* TGF-β1 (1 ng/ml).

Supplementary figure 2 Time-effect relationship of TGF-β1 on Smad3 and p-Smad3 protein expressions in myocardial fibroblasts under hypoxia conditions. MFs were inoculated in 6-well plate, and when the cells grew to 60%-70% confluence, 0.2%FBS was replaced for culture for 24 h and TGF-β1 (5 ng/ml) was added. The total protein of cells was extracted after 15, 30, 45, 60 and 90 min, respectively, for immunoblotting test. A: Representive immunoblots of protein p-Smad3 and Smad3 in MFs. B: Bars represent protein quantification of p-Smad3 and Smad3 relative to β-actin. TGF-β1, transforming growth factor-β1. Results are mean ± SD (n=3); a*P* < 0.05 vs. TGF-β1 (0 min).

Supplementary figure 3 Effect of Smad3-specific siRNA on the expression of Smad3 protein in Myocardial fibroblasts under hypoxia conditions. MFs was transfected with Smad3 siRNA using the Lipofectamine®RNAiMAX transfection reagent for 6 h, and the total cell protein was extracted by RIPA. Smad3 protein level was measured by immunoblotting. A: Representive immunoblots of protein Smad3 in MFs. B: Bars represent protein quantification of Smad3 relative to β-actin. Results are mean ± SD (n=3); **P*<0.05 *vs.* Scrambled RNA; #*P*<0.05 *vs.* Smad3 siRNA 25 nM.

Supplementary figure 4 Effects of Smad3 silencing on the TGF-β1-induced p-Smad3 nucleus translocation in myocardial fibroblasts under hypoxia conditions. MFs was transfected with Smad3 siRNA using the Lipofectamine®RNAiMAX transfection reagent for 6 h, 0.2%FCS was replaced for 24 h, and TGF-β1(5 ng/ml) was added for 60min. Total protein and nuclear fractions of the cells were extracted, respectively. Smad3/p-Smad3 levels were measured by immunoblotting. A: Representive immunoblots of protein Smad3 in MFs. B: Bars represent protein quantification of Smad3 relative to β-actin. TGF-β1, transforming growth factor-β1; Cyto-P-Smad3, p-Smad3 in the cytoplasm; Nucl-P-Smad3, p-Smad3 in the nucleus; Results are mean ± SD (n=3); **P* < 0.05 *vs.* Control; #*P*<0.05 *vs.* Scrambled RNA + TGF-β1(5 ng/ml).

Supplementary figure 5 Effect of Transfection with a Smad3-expressing plasmid on the expression of Smad3 protein in myocardial fibroblasts. MFs were transfected with a plasmid expressing Smad3 for 48 h, and the total cell protein was extracted by RIPA. The level of Smad3 was measured by immunoblotting. A: Representive immunoblots of protein Smad3 in MFs. B: Bars represent protein quantification of Smad3 relative to β-actin. Results are mean ± SD (n=3); **P* < 0.05 *vs.* Liposome.

Supplementary figure 6 Effect of Smad3 overexpression on the TGF-β1-induced p-Smad3 nucleus translocation in myocardial fibroblasts under hypoxia conditions. MFs were transfected with a plasmid expressing Smad3 for 48 h, 0.2% FCSs were replaced for 24 h, and TGF-β1(5ng/ml) was added for 60min. Total protein and nuclear fractions of the cells were extracted, respectively. Smad3/p-Smad3 levels were measured by immunoblotting. A: Representive immunoblots of protein Smad3 in MFs. B: Bars represent protein quantification of Smad3 relative to β-actin. TGF-β1, transforming growth factor-β1; Cyto-P-Smad3, p-Smad3 in the cytoplasm; Nucl-P-Smad3, p-Smad3 in the nucleus; Results are mean ± SD (n=3); **P* < 0.05 *vs.* Control; #*P* <0.05 *vs.* TGF-β1 (5 ng/ml).
